# Enhanced Fire Safety of Energy-Saving Foam by Self-Cleavage CO_2_ Pre-Combustion and Phosphorus Release Post-Combustion

**DOI:** 10.3390/molecules29153708

**Published:** 2024-08-05

**Authors:** Fengyun Sun, Lijun Wang, Tiantian Gao, Yuanyuan Zhong, Kefa Ren

**Affiliations:** 1 School of Mechanical Engineering, Chengdu University, Chengdu 610106, China; zhongyuanyuan@stu.cdu.edu.cn; 2 Clinical College, Chengdu University, Chengdu 610106, China; 15281073918@163.com (L.W.); 13219016113@163.com (T.G.); 3 College of Earth and Planetary Sciences, Chengdu University of Technology, Chengdu 610051, China

**Keywords:** rigid polyurethane foam, flame retardant, catalytic self-cleavage, thermal insulation

## Abstract

Rigid polyurethane foam (RPUF) is widely utilized in construction and rail transportation due to its lightweight properties and low thermal conductivity, contributing to energy conservation and emission reduction. However, the inherent flammability of RPUF presents significant challenges. Delaying the time to ignition and preventing flame spread post-combustion is crucial for ensuring sufficient evacuation time in the event of a fire. Based on this principle, this study explores the efficacy of using potassium salts as a catalyst to promote the self-cleavage of RPUF, generating substantial amounts of CO_2_, thereby reducing the local oxygen concentration and delaying ignition. Additionally, the inclusion of a reactive flame retardant (DFD) facilitates the release of phosphorus-oxygen free radicals during combustion, disrupting the combustion chain reaction and thus mitigating flame propagation. Moreover, potassium salt-induced catalytic carbonization and phosphorus derivative cross-linking enhance the condensed phase flame retardancy. Consequently, the combined application of potassium salts and DFD increases the limiting oxygen index (LOI) and reduces both peak heat release rate (PHRR) and total heat release (THR). Importantly, the incorporation of these additives does not compromise the compressive strength or thermal insulation performance of RPUF. This integrated approach offers a new and effective strategy for the development of flame retardant RPUF.

## 1. Introduction

As a classic thermal insulation material, rigid polyurethane foam (RPUF) is extensively utilized in rail transit and construction industries [1,2,3]. Particularly driven by “carbon peaking” and “carbon neutrality” goals, the application of RPUF has garnered increasing attention [4,5]. However, due to its cellular structure and flammable chemical elements, RPUF is highly susceptible to ignition. In recent decades, RPUF-related fires have led to significant casualties and substantial property losses. Hence, enhancing the flame retardancy of RPUF has remained a primary focus for researchers [6,7,8].

The most conventional method to flame-retard RPUF involves directly incorporating an appropriate amount of flame retardants (FRs). For example, Meng [9] et al. investigated the effect of expandable graphite (EG) and ammonium polyphosphate (APP) on the flame retardancy of RPUF. The results demonstrate that the addition of either EG or APP individually could significantly enhance the flame retardancy of RPUF. Furthermore, when EG and APP were incorporated simultaneously at a mass ratio of 1:1, the flame retardancy of RPUF reached its optimum level. However, this improvement was accompanied by a reduction in compressive strength. Thirumal [10] et al. synthesized water-blown rigid polyurethane foam (PUF) using melamine polyphosphate (MPP) and melamine cyanurate (MC) as flame-retardant additives. Their findings indicated that PUF incorporated with MC exhibited superior mechanical and thermal oxidation stability compared to PUF with MPP. Conversely, the flame retardancy of PUF containing MPP was found to be superior to that of PUF containing MC. Similar FRs, such as tris (1-chloro-2-propyl) phosphate [11], magnesium hydroxide [12], aluminum hydroxide [13] and zinc borates [14], are also added to RPUF to enhance its flame retardancy. This method is straightforward and effective, maintaining its dominance in industrial applications. However, due to the high foaming ratio of RPUF, a substantial quantity of FRs is necessary to achieve a satisfactory flame retardancy. This high addition rate, however, may disrupt the foaming balance and compromise the mechanical properties of RPUF. Currently, the research emphasis lies on minimizing the FRs dosage while simultaneously ensuring the excellent flame retardancy and mechanical properties of RPUF. One of the most common approaches is to use nanofillers to enhance the flame retardancy of RPUF. Commonly used nano flame retardants include nano-clay [15], nano-SiO_2_ [16] and carbon nanotubes [17]. Particularly, layered silicates have been widely employed to prepare flame-retardant polymers/nanocomposites due to their low cost, high aspect ratio and unique intercalation/exfoliation properties. For instance, Zheng [18] et al. combined 2 wt.% clays with reactive FRs to flame-retard RPUF, achieving a balance between mechanical and flame retardancy. The incorporation of a low percentage of clays, when combined with liquid reactive FRs, does not adversely affect the foaming process, as they do not alter the viscosity of the component A (polyols). Moreover, the clays function as a nucleating agent, enhancing the mechanical strength while the reactive FRs can ensure long-term flame retardancy. When exposed to flames, the reactive FRs release phosphorus-containing free radicals in the gas phase, halting the combustion chain reaction, while the clays strengthen the char layer in the condensed phase. However, inert clays lack gas phase flame retard ability and have limited efficacy in converting RPUF into a char layer. Therefore, enhancing the ability of fillers to catalyze carbonization could further improve the flame retardancy of RPUF.

Since CO_2_ is non-corrosive and has minimal environmental impact, it is commonly utilized as an efficient fire extinguishing agent. Although nearly all polymers generate CO_2_ during combustion, the quantity released is generally insufficient to confer flame retardancy. Modifying the traditional thermal decomposition pathway of polymer combustion to enhance CO_2_ release could potentially improve the flame retardancy of polymers. Specifically, if a substantial amount of CO_2_ is rapidly emitted during the thermal decomposition phase rather than at the onset of combustion, self-extinguishing behavior or delayed ignition may be achieved. This mechanism is critical, as it provides valuable time for evacuation and enhances fire safety.

Inspired by the study on polymer degradation using potassium salts as fillers by Fan [19] and Zhu [20] et al., which demonstrated that potassium salts can rapidly degrade polymers into char and release substantial amounts of CO_2_, this degradation mechanism evidently holds substantial potential for enhancing the flame retardancy of RPUF. However, considering that this catalytic effect primarily occurs before combustion, the catalytic action of potassium salts becomes less effective in preventing flame spread once combustion has been initiated. Therefore, this study also incorporates reactive FRs to mitigate flame propagation post-combustion. The objective of this study is to preliminarily investigate the effects of combining potassium salts with reactive FRs on the flame retardancy, mechanical properties and thermal insulation properties of RPUF. Given the low cost, minimal amount of flame retardants required and simple preparation process, this study offers a promising approach for the industrial-scale production of flame-retardant RPUF.

## 2. Results

### 2.1. Characterization of Reactive FRs

**Figure 1:**
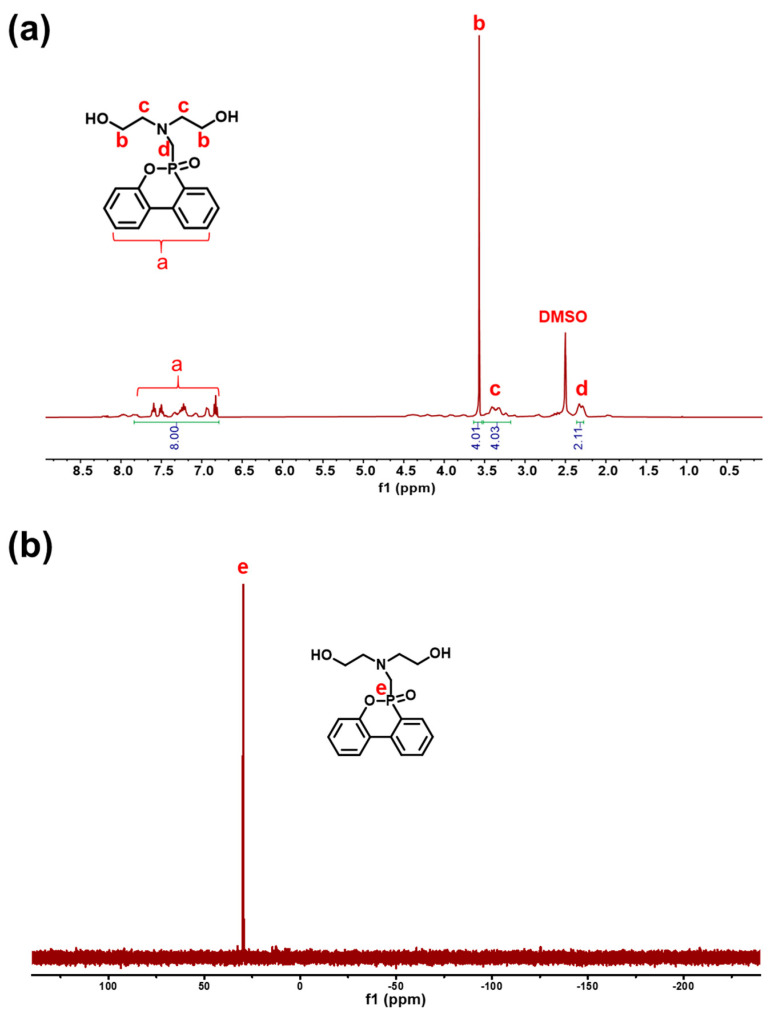
(**a**) ^1^H NMR and (**b**) ^31^P NMR spectra of DFD in DMSO-d6.

Figure 1a depicts the ^1^H NMR spectrum of DFD. In this spectrum, the peak observed at 2.50 ppm corresponds to the proton of DMSO. The peaks in the range of 6.7–7.8 ppm are attributed to the protons (***a***) on the benzene ring. The peak at 3.6 ppm is associated with the protons (***b***) on the –O–CH_2_–, while the peaks between 3.25 and 3.5 ppm correspond to the protons (***c***) on the –N–CH_2_–group. Additionally, the peaks in the range of 2.2–2.4 ppm correspond to the protons (***d***) on the –P–CH_2_–N–. The peak area ratios for (***a***), (***b***), (***c***) and (***d***) are approximately 8:4:4:2, aligning well with the theoretical molecular structure of DFD. Furthermore, Figure 1b presents the ^31^P NMR spectrum of DFD, which exhibits a single peak (***e***) at about 29 ppm, also consistent with the theoretical molecular structure of DFD [21].

**Figure 2:**
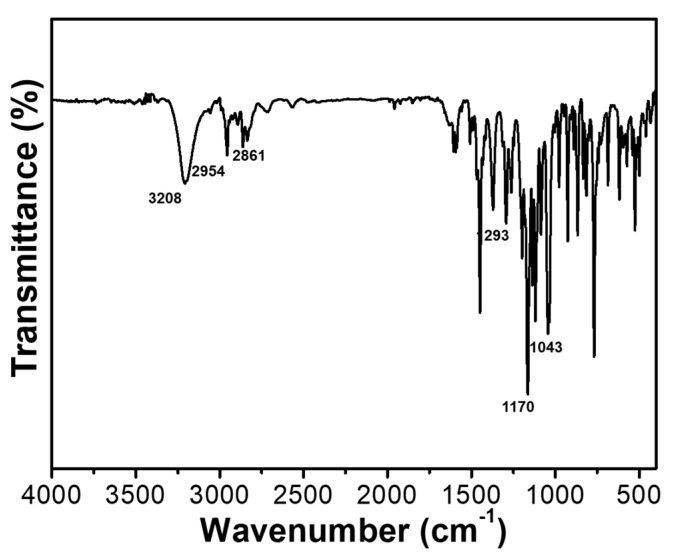
FTIR spectrum of DFD.

The chemistry structure of DFD was further characterized using FTIR spectroscopy, as depicted in Figure 2. Several characteristic peaks were observed: the peak at 3208 cm^−1^ corresponds to the –OH absorption peak, the peaks at 2954 cm^−1^ and 2861 cm^−1^ are attributed to the –CH_2_– groups and the peaks at 1293 cm^−1^, 1170 cm^−1^ and 1043 cm^−1^ correspond to P=O, P–O–C and –C–N–, respectively [22]. Additionally, the disappearance of the P–H peak at 2436 cm^−1^ further confirms the complete reaction of DOPO. Based on these results, the target product, DFD, a phosphorus-containing diol, was successfully synthesized.

### 2.2. Physical Properties of RPUFs and Flame-Retardant RPUFs

**Figure 3:**
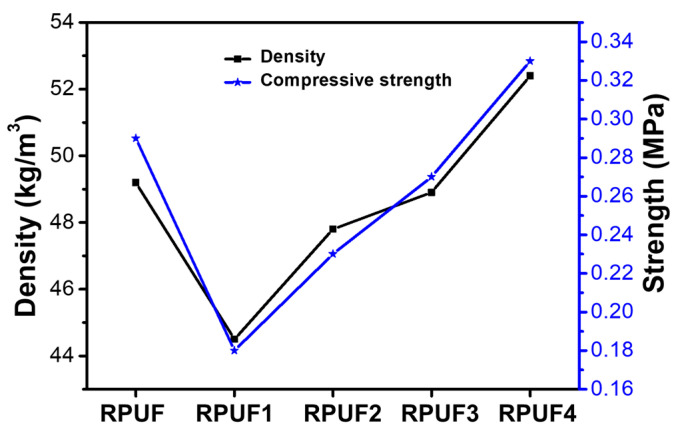
The apparent density and compressive strength of RPUF and flame-retardant RPUF.

The compressive strength and apparent density of both RPUF and flame-retardant RPUF are presented in Figure 3. Both properties exhibit a trend consistent with a power–law relationship (log (strength property) = log A + B × log (density) (where the A and B are constants)) [23]. This behavior is evidently influenced by the incorporation of FRs. The underlying mechanism is speculated as follows: potassium salt itself is an effective catalyst for the synthesis of polyurethane. However, the excessive addition of this catalyst may disrupt the foaming equilibrium. This phenomenon was observed in our experiments, where the introduction of potassium salt significantly accelerated the foaming rate. This acceleration will cause the “foaming reaction” to dominate over the “gelation reaction”, leading to a decrease in mass per unit volume and the formation of a partially open-cell structure, which subsequently reduces both density and compressive strength. In contrast, the addition of powdered solid DFD appears to act as a nucleating agent during the foaming process, promoting the formation of more closed cells. This nucleation effect enhances both the density and compressive strength of the material. When DFD is added alone, its nucleation effect is most pronounced, resulting in the highest observed values for density and compressive strength [24].

**Figure 4:**
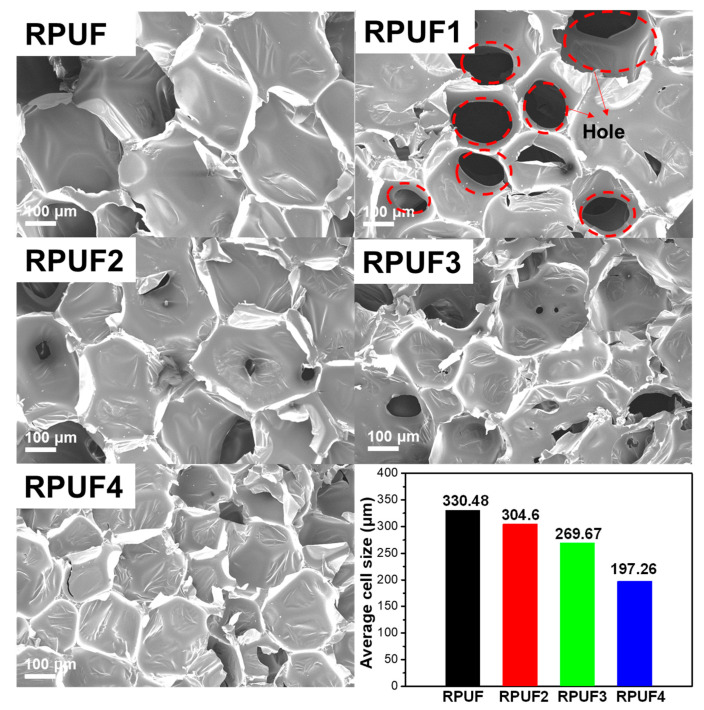
The SEM images of RPUF, RPUF1, RPUF2, RPUF3 and RPUF4, along with their average cell sizes (the pore diameter cannot be measured due to the open pore structure of RPUF1).

To further confirm the above inference, the microstructures of RPUF and flame-retardant RPUF were observed, as shown in Figure 4. Indeed, except for RPUF1, which exhibits an irregular cell structure with some destroyed cells, the other samples display a closed-cell structure. Based on the cross “×” fixed-point method, using Nano Measurer software (version number: 1.2.5) to quantify the cell diameters of closed-cell samples, it was found that RPUF has the largest cell size. With the addition of DFD, the cell size gradually decreases, with RPUF4 exhibiting the smallest cell size due to the exclusive presence of DFD. Therefore, DFD promotes an increase in sample density and an improvement in compressive strength.

### 2.3. Thermal Stability of RPUFs and Flame-Retardant RPUFs

**Figure 5:**
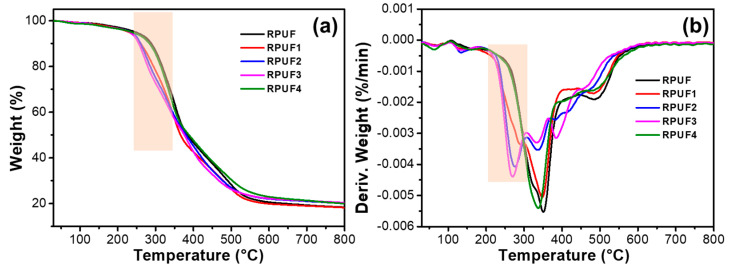
(**a**) TGA and (**b**) DTG curves of RPUF and flame-retardant RPUFs in N_2_.

To investigate the effect of potassium salt and DFD on the thermal stability of RPUFs and flame-retardant RPUFs, thermal degradation analyses were conducted using TGA, as depicted in Figure 5 and summarized in Table 1. In the case of RPUFs, the initial degradation temperature (T_5%_, defined as the temperature at which 5 wt.% thermal degradation loss occurs) is approximately 247.6 °C, marking the onset of urethane group decomposition. The maximum decomposition rate is observed at 350.6 °C (T_max_) along with a final residue of about 18.3%. Notably, the addition of potassium salt and DFD significantly alters the degradation behavior of flame-retardant RPUFs. When DFD alone is introduced, the T_5%_ of RPUF4 decreases by about 10 °C, and T_max_ decreases by about 4 °C, while the final residue increases by 1.6%. This is attributed to the phosphorus-containing units in DFD, which likely produce phosphorus-containing derivatives that promote matrix cross-linking. This degradation trend is favorable for enhancing the flame retardancy of RPUF4. However, the introduction of potassium salt results in a further reduction in T_5%_ in RPUF1, RPUF2 and RPUF3, with T_max_ decreasing by 60–80 °C. This substantial reduction is attributed to the catalytic degradation effect of potassium salt, which generates a significant amount of CO_2_, thereby delaying substrate ignition [19,20].

### 2.4. Flame Retardancy of RPUF and Flame-Retardant RPUFs

**Figure 6:**
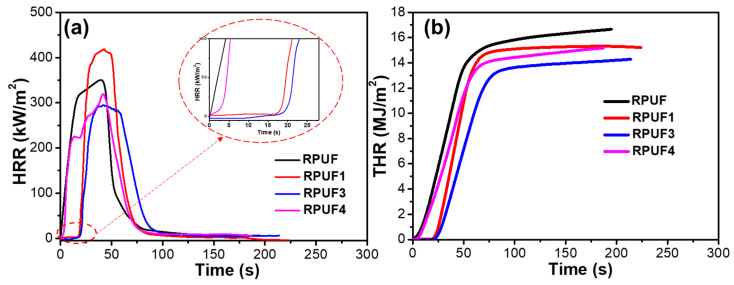
(**a**) HRR and (**b**) THR of RPUFs and flame-retardant RPUFs.

The flame retardancy of RPUFs and flame-retardant RPUFs was evaluated using UL-94 HB, LOI and CCT tests, as illustrated in Figure 6 and Table 2. The LOI value of RPUF was only 19.1%, and it did not obtain a rating in the UL-94 HB test. However, with the addition of just 2 wt.% potassium salts, the LOI value of RPUF1 increased to 23.6%, achieving the UL-94 HF2 rating, primarily due to the rapid catalytic degradation of potassium salt (as demonstrated in the thermogravimetric analysis). The substantial production of CO_2_ during this rapid thermal degradation reduced the oxygen concentration around the sample, thereby necessitating a higher oxygen concentration to sustain combustion. When only DFD was added, the LOI value of RPUF4 also increased, achieving the UL94 HF2 rating, attributable to the flame-retardant effect of phosphorus. The simultaneous addition of potassium salt and DFD further elevated the LOI of RPUF3 to 28.3%, achieving the UL-94 HF1 rating, due to the synergistic flame-retardant effects of both additives. Additionally, the large-scale combustion characteristics of RPUF and flame-retardant RPUFs were evaluated using the CCT. Upon exposure to fire, RPUF ignites immediately, with the HRR rapidly peaking (PHRR) at 353.7 kW/m^2^ and the THR reaching 16.6 MJ/m^2^. However, with the introduction of 2 wt.% potassium salt, RPUF1 releases heat after approximately 16 s and then burns rapidly, with the PHRR reaching 419.3 kW/m^2^. This increase in PHRR may be attributed to the early-stage catalytic degradation by potassium salt. When exposed to heat, potassium salt rapidly degrades the polyurethane molecular chain, producing a large amount of CO_2_ that delays immediate combustion. It can be confirmed that the average CO_2_ release of the samples containing potassium salt is significantly higher than that of the samples without potassium salt. This process, however, accumulates a significant amount of small molecular fragments that, upon reaching the ignition point, burn rapidly and release substantial heat in a short period, resulting in a PHRR of RPUF1 exceeding that of RPUF. When only DFD is added, RPUF4 exhibits a PHRR lower than that of RPUF, though it remains highly ignitable in the early stages. With the concurrent addition of DFD and potassium salt, not only is the heat release time of RPUF3 delayed, but the PHRR and THR are also reduced by 16.5% and 13.3%, respectively. Additionally, the fire performance index (FPI) (ignition time/PHRR) for RPUF1 and RPUF3 containing potassium salt was significantly higher than that for RPUF and RPUF4 without potassium salt. This enhanced flame-retardant effect is crucial for providing more evacuation time and reducing fire risk.

**Figure 7:**
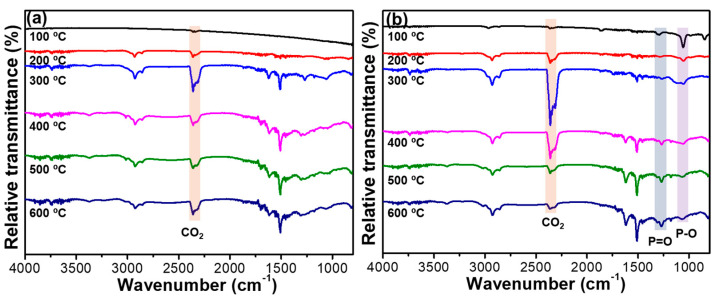
TG-FTIR spectra of (**a**) RPUF and (**b**) RPUF3.

To further elucidate the flame-retardant mechanism of potassium salt and DFD in the gas phase, TG-FTIR was employed to test two representative samples, RPUF and RPUF3. The chemical composition of their residues post-combustion was also analyzed by FTIR. As illustrated in Figure 7, RPUF exhibits distinct characteristic peaks of CO_2_ at approximately 2340 cm^−1^ as the temperature increases, with the peak intensity reaching its maximum at 300 °C due to the decomposition of the urethane groups at this temperature. In contrast, RPUF3 displays different decomposition characteristics. The intensity of the characteristic peak of CO_2_ for RPUF3 at 300 °C is significantly higher than that of RPUF, attributable to the catalytic degradation facilitated by potassium salt. The potassium salt catalyzes the degradation of urethane groups, leading to the early-stage production of substantial amounts of CO_2_, thereby enhancing the flame-retardant effect of RPUF3. Moreover, the presence of DOPO units in DFD contributes to the flame-retardant properties of RPUF3. As the temperature increases, RPUF3 exhibits P-O and P=O characteristic peaks around 1050 cm^−1^ and 1260 cm^−1^, respectively. These phosphorus-containing free radicals are advantageous for capturing H· and HO· radicals during the combustion of RPUF3, effectively terminating the chain reaction of combustion and further enhancing the flame-retardant effect in the later stages of combustion [25,26].

**Figure 8:**
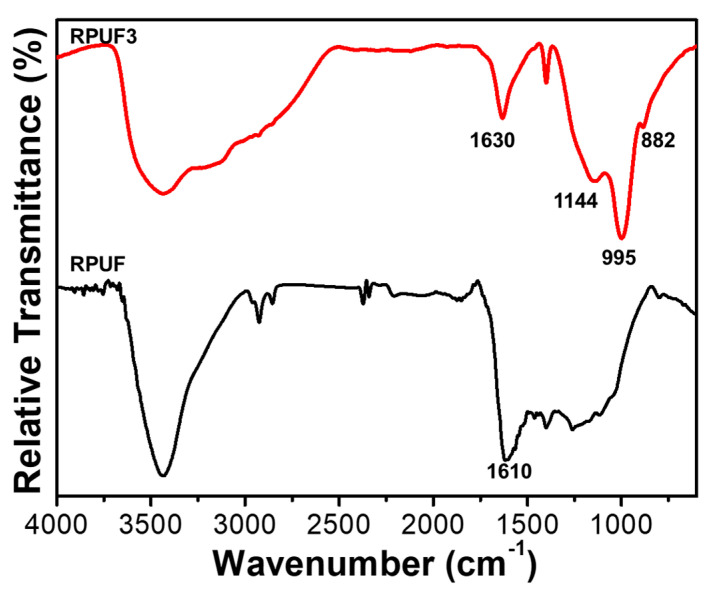
FTIR spectra of the residues after combustion of RPUF and RPUF3.

The flame-retardant mechanism in the condensed phase was elucidated by analyzing the chemical composition of the residues post-combustion. From Figure 8, RPUF displays a typical aromatic char structure with a prominent peak at 1605 cm^−1^ after combustion. In contrast, RPUF3 exhibits additional peaks. The peak at 1630 cm^−1^ is attributed to the typical C=C bond, indicative of the catalytic carbonization by potassium salt. Peaks at 882 cm^−1^, 995 cm^−1^ and 1144 cm^−1^ are attributed to DFD, corresponding to P–O–P, P–O–C and P=O bonds, respectively. These results demonstrate that potassium salt and DFD contribute to catalytic carbonization in the condensed phase, which effectively inhibits the continuous burning of the flame [27,28,29].

**Scheme 1:**
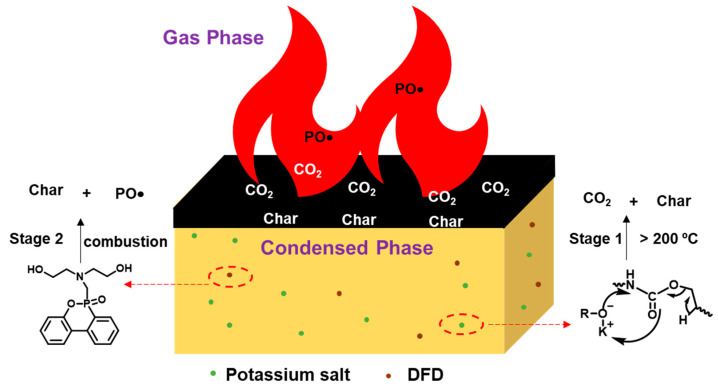
Schematic diagram of flame-retardant mechanism.

Based on the experimental results and corroborating literature [30,31,32], the flame-retardant mechanism of potassium salt and DFD is proposed as illustrated in Scheme 1. Upon exposure to flame, the temperature of RPUF3 gradually increases. When the temperature exceeds 200 °C, potassium salt rapidly catalyzes the degradation of urethane groups, releasing CO_2_. At this stage, the sample has not yet ignited, and the released CO_2_ lowers the oxygen concentration around the sample, delaying its combustion while simultaneously accumulating a significant amount of small molecular combustible fragments. Upon reaching the ignition point, the sample combusts vigorously. Concurrently, PO• radicals generated by the thermal decomposition of DFD terminate the combustion chain reaction, thereby reducing heat release and flame propagation. Additionally, the catalytic carbonization facilitated by potassium salt and DFD in the condensed phase impedes the continuous burning of the flame. Thus, this synergistic flame-retardant effect significantly enhances the fire safety of the material.

### 2.5. Thermal Insulation of RPUFs and Flame-Retardant RPUFs

**Figure 9:**
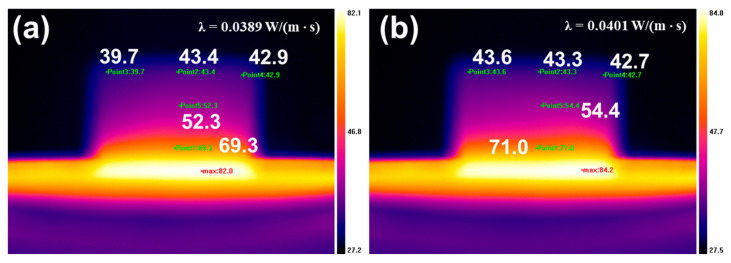
Pseudo-color thermal images of (**a**) RPUF and (**b**) RPUF3.

As an energy-saving material, the thermal insulation performance of RPUF is the most crucial indicator in practical applications. To intuitively assess the impact of flame retardants on the thermal insulation, the sample was placed on a hot plate at 85 °C for one hour, and the surface temperature of the sample was recorded using a thermal IR image tester. As shown in Figure 9, the surface temperatures of RPUF and RPUF3 are rather similar, and their thermal conductivity coefficients (λ) also show negligible differences. This indicates that the flame retardant has minimal impact on the thermal insulation properties of RPUF. This can be attributed to two main factors: first, the thermal insulation capability of RPUF primarily relies on the intrinsic properties of the material; second, the quantity of flame retardant added is small, preventing the formation of a significant heat conduction path [33,34].

## 3. Materials and Methods

### 3.1. Materials

The polyols containing catalysts, surfactants and foaming agents (component A) and isocyanates (component B) utilized in the synthesis of RPUF were sourced from Shandong Jiuyun Insulation Materials Co., Ltd. (Dezhou, China) Potassium formate (98%), dibutyltin dilaurate (DBTDL), 9,10-dihydro-9-oxa-10-phosphaphenanthrene 10-oxide (DOPO, 99%), 37% formaldehyde in water, 1,4-dioxane (99%, with 10 ppm BHT stabilizer) and diethanolamine (99%) were procured from Kelong Chemical Reagent Factory (Chengdu, China). Distilled water was prepared in-house in the laboratory.

### 3.2. Synthesis of Reactive FRs

**Scheme 2:**
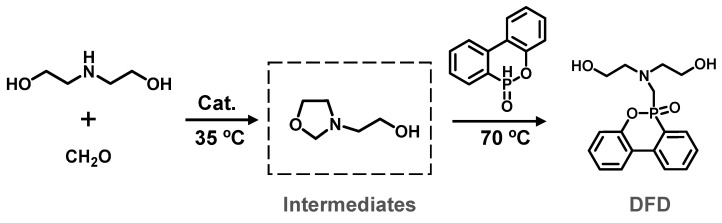
The synthesis route of DFD.

The reactive FRs, denoted as DFD, were synthesized using DOPO, formaldehyde and diethanolamine in accordance with established protocols [35,36] (Scheme 2). Initially, a mixture of 9 g of 37% formaldehyde aqueous solution and 12 g of diethanolamine was placed in a three-necked flask, with a drop of DBTDL serving as the catalyst. The reaction mixture was stirred at 35 °C for 4 h. Subsequently, the water in the mixture was removed via reduced pressure distillation, resulting in a viscous solution. A solution of 24 g of DOPO dissolved in 100 mL of 1,4-dioxane was then gradually added to the above mixture, and the reaction was continued at 70 °C for an additional 4 h. After cooling, the resulting white solid was isolated by filtration and washed three times with ethanol. The final product was dried in a vacuum oven at 100 °C for 4 h, yielding a white solid with a yield of approximately 94.0%.

### 3.3. The Prepared of RPUF and Flame-Retardant RPUFs

RPUFs and flame-retardant RPUFs were synthesized using the formulations detailed in Table 3. For the preparation of the flame-retardant RPUF, potassium salt and a reactive flame retardant were initially incorporated into component A and stirred for several minutes. Subsequently, component B was introduced, followed by immediate high-speed stirring for 15 s before being poured into a mold. The desired product was obtained through free foaming. To ensure complete curing, the foam was then placed in an oven at 80 °C for 24 h.

### 3.4. Characterization

Fourier transform infrared spectroscopy (FTIR) was conducted using a Thermo Fisher Scientific Nicolet iS20 instrument (Madison, WI, USA). ^1^H NMR and ^31^P NMR spectra were recorded on a Bruker Avance III HD 400 MHz spectrometer (Karlsruhe, Germany). Thermogravimetric analysis (TG) was carried out with a Mettler TGA/DSC1 system (Zurich, Switzerland) under a nitrogen atmosphere. Micromorphology observations were performed using a field emission scanning electron microscope (FEI-Inspect F50, Hillsboro, OR, USA). The limiting oxygen index (LOI) was measured using a Dynisco LOI instrument (Fire TestingTechnology Ltd., West Sussex, UK) in accordance with ASTM D2863-97 [37]. Horizontal burning tests (UL94 HB) were carried out on the ISO 9772–2020 [38]. Cone calorimeter tests (CCTs) were executed using an FTT cone calorimeter at a heat flux of 35 kW/m^2^, following ISO 5660-1 [38]. Apparent density was calculated as per GB/T 6343-2009 [24], with sample dimensions of 50 mm × 50 mm × 50 mm. Compressive strength testing was performed on a universal testing machine in accordance with GB/T 8813–2008 [18]. TG-FTIR analysis was conducted using a Netzsch 209F3 (Bavaria, Germany) and Bruker TENSOR27 (Karlsruhe, Germany). at a heating rate of 10 °C/min in N2 from 30 to 800 °C. Thermal conductivities of various samples were measured using a thermal conductivity meter TC3000E (Xi’an Xiaxi Electronic Technology Co., Ltd., Xi’an, China). Thermal insulation properties were recorded using IR thermal imaging cameras.

## 4. Conclusions

Based on the principle of delaying the ignition time of the material and preventing flame propagation post-combustion, this study investigates the flame retardancy of RPUF using a combination of 2 wt.% potassium salt and 10 wt.% reactive phosphorus-containing DFD. The results indicate that the inclusion of 2 wt.% potassium salt catalyzes the self-cleavage of RPUF, producing substantial quantities of CO_2_ at temperatures exceeding 200 °C, thereby delaying the ignition time of the RPUF. After combustion, the phosphorus-containing free radicals released by DFD inhibit flame spread. Additionally, the catalytic carbonization facilitated by the potassium salt and the cross-linking of phosphorus-containing derivatives decomposed by DFD contribute to the flame-retardant effects in the condensed phase. Overall, the synergistic application of these two flame retardants resulted in a 48.2% increase in LOI, a 16.5% reduction in PHRR, a 13.3% reduction in THR and the successful achievement of the UL94 HF1 rating. Furthermore, the flame-retardant strategy presented in this study may inspire future research, guiding researchers to explore combinations of other metal salts (such as iron salts, manganese salts and cobalt salts) with new phosphorus-containing flame retardants to achieve more efficient flame retardancy. Given the low cost and straightforward preparation process, this study presents a promising approach for the industrial-scale production of flame-retardant RPUF.

## Figures and Tables

**Table 1:** The formulation of RPUFs and flame-retardant RPUFs.

Samples	T_5%_ (°C)	T_max_ (°C)		Residues (%)
		T_max1_	T_max2_	
RPUF	247.6	/	350.6	18.3
RPUF1	229.3	292.3	347.3	18.2
RPUF2	233.0	275.6	336.3	20.1
RPUF3	231.3	269.3	333.6	20.4
RPUF4	237.3	/	347.0	19.9

**Table 2:** Data reflecting the flame retardancy of RPUFs and flame-retardant RPUFs.

Samples	LOI (%)	UL 94 (HB)	PHRR (kW/m^2^)	THR (MJ/m^2^)	av-CO_2_ (kg/kg)	FPI (m^2^s/kW)
RPUF	19.1	NR	353.7	16.6	1.5	<0.001
RPUF1	23.6	HF2	419.3	15.2	2.6	0.038
RPUF3	28.3	HF1	295.5	14.2	2.3	0.068
RPUF4	25.4	HF2	320.1	15.1	1.8	0.006

**Table 3:** The formulation of RPUF and flame-retardant RPUF.

Samples	Component A (g)	Component B (g)	Potassium Salt (g)	DFD (g)
RPUF	50	50	0	0
RPUF1	49	49	2	0
RPUF2	46.5	46.5	2	5
RPUF3	44	44	2	10
RPUF4	45	45	0	10

## Data Availability

Data are contained within the article.
